# Anti-*Toxoplasma gondii* antibodies in pregnant women and their newborn infants in the region of São José do Rio Preto, São Paulo, Brazil

**DOI:** 10.1590/S1516-31802011000400010

**Published:** 2011-05-05

**Authors:** Cinara de Cássia Brandão de Mattos, Lígia Cosentino Junqueira Franco Spegiorin, Cristina da Silva Meira, Thaís da Costa Silva, Ana Iara da Costa Ferreira, Fabiana Nakashima, Vera Lúcia Pereira-Chioccola, Luiz Carlos de Mattos

**Affiliations:** I MSc. Doctoral student in Health Sciences, Immunogenetics Laboratory, Department of Molecular Biology, Faculdade de Medicina de São José do Rio Preto (Famerp), São José do Rio Preto, São Paulo, Brazil.; II MD, MSc. Doctoral student in Health Sciences, Department of Gynecology and Obstetrics, Faculdade de Medicina de São José do Rio Preto (Famerp), Hospital de Base, Fundação Faculdade Regional de Medicina (Funfarme), São José do Rio Preto, São Paulo, Brazil.; III MSc. Doctoral student in Sciences, Instituto Adolfo Lutz, São Paulo, Brazil.; IV MSc. Immunogenetics Laboratory, Department of Molecular Biology, Faculdade de Medicina de São José do Rio Preto (Famerp), São José do Rio Preto, São Paulo, Brazil.; V PhD. Scientific Researcher, Laboratory of Molecular Biology of Parasites, Instituto Adolfo Lutz, São Paulo, Brazil.; VI PhD. Full Professor, Immunogenetics Laboratory, Department of Molecular Biology, Faculdade de Medicina de São José do Rio Preto (Famerp), São José do Rio Preto, São Paulo, Brazil.

**Keywords:** Toxoplasma gondii, Serologic tests, Pregnancy, high-risk, Prenatal diagnosis, Neonatal screening, Toxoplasma gondii, Testes sorológicos, Gravidez de alto risco, Diagnóstico pré-natal, Triagem neonatal

## Abstract

**CONTEXT AND OBJECTIVE:**

Toxoplasmosis transmission during pregnancy can cause severe sequelae in fetuses and newborns. Maternal antibodies may be indicators of risk or immunity. The aim here was to evaluate seropositivity for anti*-Toxoplasma gondii* (anti-*T. gondii)* immunoglobulin M (IgM) and immunoglobulin G (IgG) antibodies and IgG avidity in pregnant women and their newborn infants.

**DESIGN AND SETTING:**

Cross-sectional study in a high-risk pregnancy outpatient clinic.

**METHODS:**

Serum samples from pregnant women (n = 87) and their respective newborns (n = 87) were evaluated for anti-*T. gondii* antibodies using indirect immunofluorescence (IIF) (IgM and IgG), enzyme-linked immunosorbent assay (ELISA) (IgG) and an avidity test.

**RESULTS:**

Anti-*T. gondii* antibodies were identified in 64.4% of the serum samples from the mothers and their infants (56/87). Except for two maternal serum samples (2.3%), all others were negative for anti-*T. gondii* IgM antibodies, using IIF. The results showed that 92.9% of the pregnant women had high IgG avidity indexes (> 30%) and four samples had avidity indexes between 16 and 30%. Two women in the third trimester of pregnancy were positive for anti-*T. gondii* IgM antibodies; their babies had avidity indexes between 16 and 30%. The avidity indexes of serum from the other 83 newborns were similar to the results from their mothers.

**CONCLUSIONS:**

The results showed that 2% of the pregnant women were at risk of *T. gondii* transmission during the gestational period. These data seem to reflect the real situation of gestational toxoplasmosis in the northwestern region of the state of São Paulo.

## RESUMO

**CONTEXTO E OBJETIVOS:**

A toxoplasmose, quando transmitida durante a gestação, pode causar graves sequelas em fetos e neonatos. Anticorpos maternos podem ser indicadores de risco ou de imunidade. O objetivo foi avaliar a positividade dos anticorpos das classes imunoglobulina M (IgM) e imunoglobulina G (IgG) anti-*Toxoplasma gondii* (anti-*T. gondii*)*,* bem como a avidez de IgG em gestantes e seus neonatos.

**TIPO DE ESTUDO E LOCAL:**

Estudo transversal em ambulatório de gestação de alto risco.

**MÉTODOS:**

Anticorpos *anti-T. gondii* foram avaliados em amostras de soro de gestantes (n = 87) e seus respectivos neonatos (n = 87) com o uso dos métodos imunofluorescência indireta (IFI) (IgM e IgG), ensaio imunoenzimático (ELISA) (IgG) e avidez.

**RESULTADOS:**

Anticorpos anti-*T. gondii* foram identificados em 64,4% das amostras de soro das mães e seus bebês (56/87). Com exceção de duas amostras de soro materno (2,3%), todas as demais foram negativas anticorpos IgM anti-*T. gondii* determinado pela IFI. Os resultados mostraram que 92,9% das gestantes tinham índices elevados de avidez de IgG (> 30%) e 4 amostras apresentaram índices de avidez entre 16-30%. Duas gestantes no terceiro trimestre da gravidez eram positivas IgM anti-*T. gondii*; seus bebês apresentaram índices de avidez entre 16 e 30%. Os índices de avidez dos soros dos outros 83 recém-nascidos foram semelhantes àqueles encontrados nas amostras maternas.

**CONCLUSÕES:**

Os resultados mostraram que 2% das gestantes estavam sob risco de transmissão de *T. gondii* durante o período gestacional. Estes dados parecem refletir a real situação da toxoplasmose gestacional na região noroeste do Estado de São Paulo.

## INTRODUCTION

**Figure 1:**
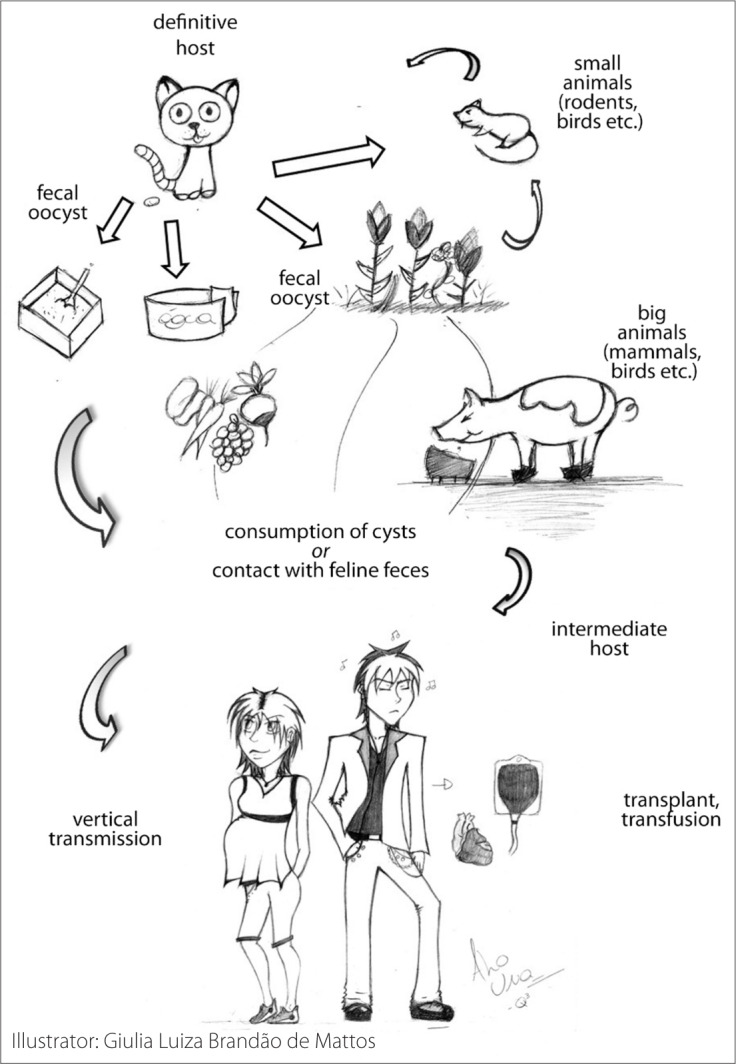
*Toxoplasma gondii* life cycle and human infection.

Toxoplasmosis is a zoonosis caused by *Toxoplasma gondii,* an obligate intracellular protozoan parasite within the apicomplexa classification that can infect many different species of mammals and birds. Humans can be infected by consumption of raw or undercooked red meat or vegetables, unpasteurized milk or contaminated water, or after contact with cat feces.^1^ Additionally, fetuses can be infected by transplacental transmission, a condition that may cause significant sequelae in babies. The life cycle of toxoplasmosis is shown in Figure 1.

During acute infections, the parasites differentiate within tissue cysts in the muscles and brain. Most primary infections are asymptomatic and, in fact, only 10-20% of all patients infected by *T. gondii* are symptomatic. In these cases, toxoplasmosis can be a serious public health problem.^2,3^

Fetuses of women acutely infected during pregnancy may present with severe damage, which also constitutes an important public health problem due to the resulting high morbidity and mortality rates. Most congenitally infected newborn babies have no clinical signs but are at risk of developing retinochoroiditis during childhood or adolescence.^4^

The risk of fetal contamination and the severity of sequelae depend on the stage of pregnancy at which the mother becomes infected.^4,5^ Early in pregnancy, infections are less likely to cross the placental barrier, but when this does occur the consequences are more serious. In general, when infection occurs late in pregnancy, babies have mild symptoms or are asymptomatic at birth.^6–8^ High parasite counts in the amniotic fluid are associated with severe outcomes.^8^ However, time of infection during pregnancy is not the only factor that contributes towards the different outcomes, since parasitic virulence is also important with regard to the severity of the disease.^9^

Different studies have reported the seroprevalence of toxoplasmosis in pregnant women and newborns in different regions of the world, including South America.^10^ Additionally, Brazilian studies have demonstrated that infection rates among pregnant women vary according to the geographical region.^10–16^ However, there are only a few studies evaluating anti-*T. gondii* antibodies and their avidity in mothers and their newborns in the state of São Paulo. Further studies that analyze anti-*T. gondii* antibodies in paired mother-baby serum samples may contribute towards better understanding of congenital toxoplasmosis in specific regions.^15,17^

## OBJECTIVES

**Figure 2:**
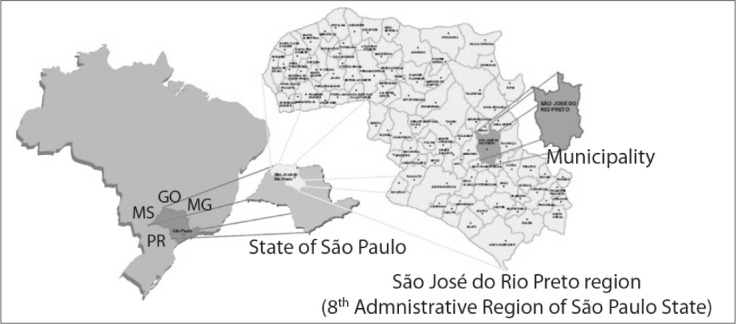
Map of Brazil indicating location of state of São Paulo and map of São José do Rio Preto region including 96 municipalities.^18^

The aim of this study was to evaluate the seropositivity of pregnant women and their newborn infants for anti*-T. gondii* immunoglobulin M (IgM) and immunoglobulin G (IgG) antibodies. The pregnant women were attended at a reference outpatient clinic for high-risk pregnancies in São José do Rio Preto. This region, located in the northwest of the state of São Paulo, is composed of 96 municipalities with a population of around 1.5 million (Figure 2).^18^

## METHODS

### Clinical samples

This cross-sectional descriptive study analyzed the positivity of 174 serum samples for anti-*T. gondii* IgM and IgG antibodies and the avidity of IgG antibodies. From May 2005 to June 2007, 87 serum samples were collected from consecutive pregnant women at different gestational ages as follows: (i) 13 women in the first trimester; (ii) 36 women in the second trimester; and (iii) 38 women in the third trimester. Another 87 samples were collected from the babies’ umbilical cords at birth. All the pregnant women selected for this study were considered as having “high-risk pregnancy”, as determined by the Ministry of Health's policy.^19^ They were attended and gave birth at the high-risk gestational outpatient clinic of the teaching hospital (Hospital de Base) of Fundação Faculdade Regional de Medicina (Funfarme), São José do Rio Preto, state of São Paulo, Brazil. This is a tertiary-care regional reference center. The number of samples evaluated represented 23.7% of the total number (n = 367) of high-risk pregnant women attended during the period of this study, as reported in our previous paper.^20^

After blood collection (around 5 ml) from mothers and from the umbilical cords, the samples were immediately sent to the Immunogenetics Laboratory of the Department of Molecular Biology, Faculdade de Medicina de São José do Rio Preto (Famerp). The serum samples were stored at −20 °C until use. All samples were assayed by means of indirect immunofluorescence (IIF) (for IgM and IgG), enzyme-linked immunosorbent assay (ELISA) (for IgG) and an avidity test. All the pregnant women gave their written consent for the procedures and the institution's Ethics Committee approved this study (case number 295/2008).

### *Toxoplasma gondii* and antigens

*T. gondii* *RH* strain tachyzoites were grown and maintained in the ascites of Swiss mice by means of intraperitoneal inoculation. At three to four-day intervals after infection, peritoneal fluid from each mouse was collected in phosphate-buffered saline (PBS) solution at pH 7.2. The mixture was centrifuged at 1,000 g for 10 minutes. The sediment containing the parasites was washed twice in PBS, the parasites were counted and the concentration was determined in order to prepare the antigens. For IIF antigens, the centrifuge pellets were suspended in PBS at a concentration of 2 x 10^7^ cells/ml. The tachyzoites were incubated in 2% buffered formalin for 30 minutes at 37 °C, washed twice in PBS, centrifuged at 1,000 g for 10 minutes and finally fixed on glass slides. For ELISA, the crude extract of tachyzoites was obtained as previously described.^21^ The parasites were sonicated (10 cycles of 1.0 A/minute for five minutes with two-minute intervals). Subsequently, the aliquots were dissolved in 0.3 M NaCl (sodium chloride) and the protein concentration was determined in a Nanodrop ND1000 spectrophotomer.

### Serological reactions

IIF was carried out as previously described,^22^ in order to determine whether anti-*T. gondii* IgG and IgM antibodies were present or absent. The samples were used in serial dilutions and assayed in duplicate. The dilutions went from 1:4 to 1:4096, and the cutoff point was determined as 1:16. For ELISA and the *Toxoplasma*-specific IgG avidity assay, the samples were assayed in duplicate at a dilution of 1:500. The optical density (OD) cutoff for ELISA at a wavelength of 492 nm was 0.190. The *Toxoplasma*-specific IgG avidity assay was performed as previously described.^22^ The basic ELISA test was used except that: (i) each serum sample was analyzed in two fourfold titration rows at a dilution of 1:500; (ii) after one hour of incubation at 37 °C, the first row was washed three times with 250 ml of 6 M urea in PBS containing 0.05% Tween 20, in order to remove low-avidity antibodies from their binding sites. The control row was washed three times using the buffer without urea. The formula to calculate the IgG avidity index was: OD values under dissociative conditions/OD values of control without urea x 100.

A low avidity index (up to 15%) was indicative of an infection within the previous five months; an avidity index between 16 and 30% was indicative of an infection more than five months ago; and a high avidity index (over 30%) represented chronic infection. For ELISA, the absorbance values were subtracted from the background, and the arithmetic mean was calculated. The cutoff was calculated for each reaction using a serum panel from 20 healthy individuals (data not shown).

### Statistical analysis

Fisher's exact test was used to evaluate associations in the serological analysis, between maternal and newborn samples.

## RESULTS

Among the 87 pregnant women evaluated, 43.7% (n = 38) were Caucasians, 44.8% (n = 39) were of mixed race, 10.3 (n = 9) were blacks and 1.2% (n = 1) were Amerindians. The mean age and gestational age were 27.5 years (± 6.9) and 25.5 weeks (± 8.4), respectively.

Anti-*T. gondii* IgG antibodies, as determined by ELISA and IIF, were identified in 64.4% (56/87) of both the maternal and the umbilical cord serum samples. The samples from the other 31 pregnant women and their babies (35.6%) were negative for toxoplasmosis. All the maternal serum samples except for two (2.3%) were negative for anti-*T. gondii* IgM antibodies, as determined by IIF. In both of these cases, the antibodies were detected in the pregnant women during the third trimester of gestation. However, IgM antibodies were not isolated in the serum of the newborns, since fetuses are unable to produce IgM antibodies.^14^ These two pregnant women did not give their consent for amniotic fluid to be collected and therefore the fetal infection could not be confirmed by means of the polymerase chain reaction (PCR).

The results showed that 92.9% (52/56) of the pregnant women infected with *T. gondii* had high avidity indexes for IgG antibodies (≥ 30%). Samples with avidity of less than 15% were not found. However, four serum samples had avidity indexes between 16 and 30%. Of these, two samples were from pregnant women in the third trimester of pregnancy who were positive for anti-*T. gondii* IgM antibodies, and their babies had avidity indexes between 16 and 30%. The other two women were in their second trimester of gestation and their babies presented avidity indexes of up to 30%. The avidity indexes of the other serum samples from the umbilical cords were similar to those found in their mothers. These results are shown in detail in Table 1. The results from the maternal and newborn serological analyses were not statistically significant (IIF/ELISA IgG: P = 1.000; IgG avidity: P = 0.6788).

**Table 1.** Determination of anti-*Toxoplasma gondii* antibodies in maternal and umbilical cord serum samples using indirect immunofluorescence (IIF), enzyme-linked immunosorbent assay (ELISA) and an immunoglobulin G (IgG) avidity test, São José do Rio Preto, state of São Paulo, Brazil

	IIF/ELISA IgG* (n = 174)		IIF IgM (n = 87)		IgG avidity† (n = 174)		
	Negative	Positive	Negative	Positive	≤ 15%	16-30%	≥ 30%
Newborn	31	56	Not determined	Not determined	0	2	54
Maternal	31	56	85	2	0	4	52
1^st^ trimester	4	9	13	0	0	0	9
2^nd^ trimester	14	22	36	0	0	2	20
3^rd^ trimester	13	25	36	2	0	2	23

* P = 1.000

† P = 0.6788 (calculated by means of Fisher's exact test)

IIF = indirect immunofluorescence; ELISA = enzyme-linked immunosorbent assay; IgG = immunoglobulin G; IgM = immunoglobulin M.

## DISCUSSION

Since toxoplasmosis is highly prevalent in Brazil and causes serious problems during pregnancy,^10^ we decided to investigate the serum status of a group of high-risk pregnant women with regard to anti-*T. gondii* IgM and IgG antibodies. These patients were attended and their babies were born at a high-risk pregnancy outpatient clinic in São José do Rio Preto. Our results showed that 64.4% of the women with high-risk pregnancies had toxoplasmosis. These data suggest that the rate of positive findings of *T. gondii* infection was high in this group and thus corroborate other studies from the same region of the state of São Paulo^20,23^ and from other Brazilian states.^15–17,24–28^ The similarities between this and other studies carried out in some Brazilian states^15–17,28^ may reflect homogeneity regarding the laboratory diagnostic strategies used.

The avidity index helps to identify the acute phase of infections by this parasite.^29^ In this study, the majority of the infected women (92.9%) were in the chronic phase of infection (avidity indexes higher than 30%). Only anti-*T. gondii* IgG antibodies with high avidity were detected in serum samples from their babies. Since the avidity indexes were identical to those of the maternal serum and the methods used in this study were unable to differentiate IgG antibodies from mothers and babies, it can be assumed that the antibodies presented by the newborns originated from the mothers. Therefore, the majority of the pregnant women evaluated in this study seemed to present a protective level of humoral immunity against *T. gondii*, without a risk of congenital transmission. Anti-*T. gondii* IgM antibodies were identified in 2.3% of the pregnant women. These antibodies were detected in the third trimester of gestation. Simultaneously, anti-*T. gondii* IgG had avidity indexes between 16 and 30%. The data suggest that these women probably became infected around five months prior to testing; in other words, within the first trimester. When primary maternal infection occurs in this period, around 15% of the fetuses can become infected.^13,14,16^ The fetuses of these two pregnant women were probably not infected during gestation, although this condition is not conclusive. There have been reports that 70% of newborns infected during gestation do not present symptoms at birth.^30^ Additionally, around 30% of newborns do not demonstrate serological evidence of congenital infection at birth, even when the mothers present with IgM antibodies.^4,14^

Since these data demonstrate that 2.3% of the pregnant women became infected during gestation, it can be assumed that this is the level of risk of congenital transmission of *T. gondii* in the northwestern region of the state of São Paulo. This figure corroborates our additional observations.^23^ These observations highlight the importance of early diagnosis and good-quality methodology for evaluating pregnant women and newborn babies in healthcare services. This, together with the risks implicit in congenital transmission, emphasizes the need for continuous educational programs and constant monitoring of pregnant women from regions where the prevalence of infection by this parasite is high.

Despite the small number of serum samples evaluated, the results from this study shed some light on the clinical importance of combined mother-newborn evaluation using serological methods to detect not only IgM and IgG anti-*T. gondii* antibodies but also IgG avidity. Furthermore, these results draw attention to the need to investigate patient samples consisting of larger numbers of mother-newborn pairs, given the epidemiological importance of toxoplasmosis.

## CONCLUSIONS

This study demonstrated that 64.4% of the pregnant women in the northwestern region of the state of Sao Paulo became infected with *T. gondii* before pregnancy and that most of them had immune protection with high avidity indexes. Nonetheless, the study suggests that an epidemiologically significant proportion of the fetuses may be at risk of congenital transmission of *T. gondii*.
